# Visuo-perceptual Abilities in Children With Perinatal Arterial Ischemic Stroke and Associated White Matter Microstructure

**DOI:** 10.1177/08830738251394438

**Published:** 2025-11-27

**Authors:** Meghan Maiani, Nisha Lahey, Martin Bardhi, Joseph Floer Garrido, Alicia Hilderley, Helen Louise Carlson, Adam Kirton

**Affiliations:** 1 Department of Pediatrics and Clinical Neurosciences, 70401 University of Calgary, Calgary, Alberta, Canada; 2 Alberta Children's Hospital Research Institute, 2129 University of Calgary, Calgary, Alberta, Canada; 3 Hotchkiss Brain Institute, 2129 University of Calgary, Calgary, Alberta, Canada

**Keywords:** diffusion-weighted imaging, neurodevelopment, perinatal stroke, tractography, visual perception

## Abstract

Visuoperceptual skills are crucial for childhood development yet remain poorly understood in children with perinatal arterial ischemic stroke (PAIS). This study examined visuoperceptual performance and structural connectivity of visual pathways in children with perinatal arterial ischemic stroke and typically developing controls. Participants completed the Motor-Free Visual Perceptual Test (MVPT), Apples Test, Jerry John's Basic Reading Inventory (BRI), and diffusion-weighted magnetic resonance imaging (MRI) with tractography of optic pathways. Visuoperceptual and reading scores were compared by group and lesion laterality. Twenty-six perinatal arterial ischemic stroke and 27 typically developing controls were recruited. Perinatal arterial ischemic stroke children scored significantly lower on the Motor-Free Visual Perceptual Test and Jerry John’s Basic Reading Inventory and took longer on the Apples Test. Right hemisphere lesions were associated with greater deficits in visuoperceptual and reading than left-sided lesions. No correlations were found between white matter microstructure and visuoperceptual scores. Findings highlight the need for early visuoperceptual screening in perinatal arterial ischemic stroke, regardless of motor status, and further investigation by lesion laterality.

## Introduction

Visuoperceptual skills are essential for typical development, enabling children to perform fundamental actions such as reach, grasp, and ambulation.^1,2^ Visual perception is the ability to understand and interpret the information that we see, and includes subfunctions such as visual search, visual memory, and object recognition. When visuoperceptual impairments are attributed to damage of the post-chiasmic structures of the brain, this is known as cerebral visual impairment.^3^ Cerebral visual impairment can occur following perinatal arterial ischemic stroke, a focal neurologic injury occurring between fetal life and 28 days of life,^4^ but prevalence and severity are unknown. Perinatal arterial ischemic stroke often, but not always, leads to unilateral movement impairments and a diagnosis of cerebral palsy. Perinatal arterial ischemic stroke may also result in cognitive and behavioral challenges, epilepsy, and cerebral visual impairment.^5,6^ Cerebral visual impairment has been associated with learning disabilities,^7^ poorer school performance,^8^ and decreased visuomotor integration^1^ in children with cerebral palsy. Enhancing our understanding of cerebral visual impairment after perinatal stroke and associated brain differences may inform future diagnostic practices and interventions.

Children with cerebral palsy typically perform worse than their peers on visuoperceptual tasks.^9–11^ Recruiting participants to research studies based on a diagnosis of cerebral palsy can be a limitation, as cerebral palsy can be caused by heterogenous etiologies. Studying cerebral palsy as a group also excludes children with intact motor function from injuries such as perinatal arterial ischemic stroke, who may be experiencing visuoperceptual, cognitive, or behavioral dysfunction.^6^ By contrast, studying children with perinatal arterial ischemic stroke whose lesions are homogenous in timing and location leads to less within-group variability and is potentially more inclusive of those with other deficits. Recent literature has emphasized the importance of considering the specific underlying etiologies and patterns of brain damage to better understand and predict clinical outcomes related to visual functioning to facilitate earlier interventions.^11,12^ As a focal injury of defined timing in an otherwise healthy brain, perinatal arterial ischemic stroke represents an ideal human model in which to study developmental plasticity following early injury.^13^ Increased understanding of visuoperceptual impairment specific to children with perinatal arterial ischemic stroke is the first step to developing tailored assessment practices in children with cerebral visual impairment secondary to early life brain injuries.

The primary visual pathway transmits information from the retina to the visual cortex where it is then distributed, primarily to temporal and parietal lobes that guide our ability to engage with our environment. Disruptions to primary visual pathway microstructure can be measured in vivo using diffusion weighted imaging and tractography and has been described in a multitude of conditions in both adult and pediatric populations.^14,15^ These differences have been associated with visual perception, such as visual search skills.^16,17^ Perinatal stroke is known to cause altered white matter microstructure of the optic tract and optic radiations,^18–21^ with a reported 28% to 50% prevalence of visual field defects in children with perinatal arterial ischemic stroke. Our current understanding of visuoperceptual impairments and their association with structural connectivity of primary optic pathways in children with perinatal arterial ischemic stroke is limited. We hypothesize that white matter microstructure in children with perinatal arterial ischemic stroke will be associated with impaired visuoperceptual performance. The current study explored associations between the microstructural alterations in afferent visual pathways with performance on tasks of visual and perceptual functioning to address the gap in current literature and further our understanding of visual development after early life injury.

## Methods

### Participants

Twenty-six children with perinatal arterial ischemic stroke aged 7-18 years were recruited through the Alberta Perinatal Stroke Project,^22^ a population-based cohort of children with perinatal stroke, in 2022 and 2023 as part of a larger study on visual system structure and function. Data collection concluded in fall 2023 due to magnetic resonance imaging (MRI) scanner maintenance. Inclusion criteria were age 6-18 years, term birth, and an MRI-confirmed diagnosis of unilateral perinatal arterial ischemic stroke (both neonatal and arterial presumed perinatal ischemic stroke were included) for children in the stroke group. Lesion location was classified using previously described methods.^23^ Perinatal arterial ischemic stroke participants with and without sensorimotor impairments were recruited and were classified using the Pediatric Stroke Outcome Measure (PSOM),^24^ where a score >0 on the motor subscale indicated the presence of sensorimotor challenges. Exclusion criteria included neurologic disorders not attributed to stroke, the inability to participate in testing protocols, and active participation in intensive therapies. Children younger than 6 years were excluded because of the ongoing maturation of the visual system during this time. Twenty-seven age- and sex-matched typically developing controls (TDC) with no neurologic disorders were recruited through a community-based healthy control program (www.hiccupkids.ca). This study was approved by the University of Calgary Research Ethics Board (REB22-0303). Written consent and assent were obtained. Participants had the option to complete one 3-4-hour session or 2 separate visits of 1.5-2 hours each.

### Image Acquisition

MRI was performed using a 3-Tesla (T) GE MR750w research-dedicated scanner at the Alberta Children's Hospital. Participants were offered the opportunity to engage in a practice session in a mock scanner. Participants selected a movie to watch for the duration of the scan to increase comfort and reduce head motion.^25^ T1-weighted images were collected in the axial plane (166 slices, 1-mm isotropic voxels, repetition time (TR) / echo time (TE) = 8.5/3.2 ms, flip angle = 11^o^, duration = 4:53 minutes). Multi-shell diffusion-weighted images (60 directions with b = 0.2000 s/mm^2^; and 20 directions with b = 0.900 s/mm^2^) were acquired in the axial plane (2.2 mm isotropic voxels, TR/TE = 15 000/73 ms, 60 slices).

### Image Processing

Diffusion image pre-processing was completed using MRtrix3, SPM, FSL, and Synb0.^26–30^ Multishell b2000 and b900 images were concatenated prior to denoising and Gibbs ringing removal. The 60-direction b0 image was used to create a synthetic, undistorted reverse phase encoded b0 scan using Synb0,^28,29^ enabling echo planar image distortion correction and motion correction via FSLs *topup* and *eddy.*^27^ Anatomical segmentations were performed on the T1 image using SPM12, and resulting gray matter, white matter, and cerebrospinal fluid masks were combined to create a brain mask estimation.^30^ Using the multishell, multitissue constrained spherical deconvolution model, fiber orientation distribution maps were calculated following response function estimation.^26^ Remaining processing steps included bias field correction, intensity normalization, calculation of the tensor, and generation of microstructure metric maps.^26^

### Tractography

**Figure 1.**
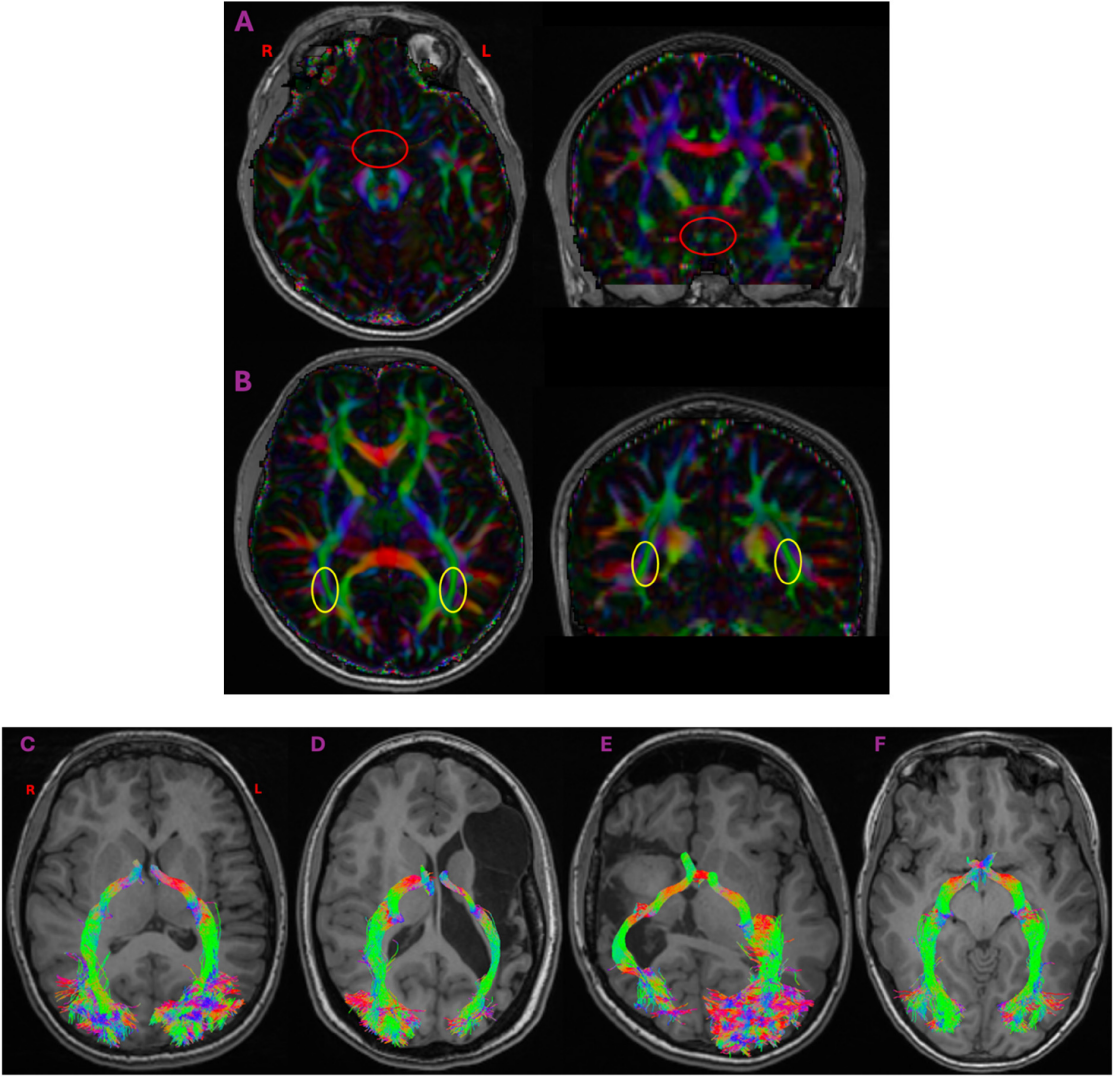
Top: T1-weighted images with a colourmap overlay in axial (left) and coronal (right) views. ROIs located posterior to the optic chiasm (A) and the posterior thalamic radiations (B) were used to generate white matter representations. Bottom: T1-weighted images and reconstructed white matter representations of the optic tracts and optic radiations in 4 participants: 2 with left-hemisphere lesions (C, D), 1 with a right hemisphere lesion (E) and 1 typically developing child (F).

Probabilistic anatomically constrained white matter tractography was performed using MRtrix3.^31^ White matter tracts were reconstructed by defining 2 regions of interest (ROIs) in the coronal plane. The first ROI was placed posterior to the optic chiasm and the second was placed on the posterior thalamic radiation of the ipsilateral hemisphere based on validated methods that generate the entire post-chiasmic tract (Figure 1).^20^ The *tckgen* command was used to reconstruct white matter representations of the optic tract and optic radiations between the 2 regions using the iFOD2 probabilistic algorithm (maximum 3000 streamlines, maximum angle 45 °, step size 1.25 mm).^20^ Using the resulting white matter streamline binary masks, white matter metrics of mean fractional anisotropy, mean diffusivity, axial diffusivity, and radial diffusivity were extracted.

### Perceptual Assessment

Assessments were selected based on several factors including clinometric properties for a pediatric population, high interrater reliability, and time required for test administration. Administration of the assessments was completed by a registered occupational therapist (M.M.). Corrected or uncorrected visual acuity measured binocularly using a Snellen chart at a threshold of 20/30 was required to participate in the assessments. Visual field testing results were obtained with participant consent from the medical chart where available. Participants were free to take breaks at any point between the different tasks.

The Motor-Free Visual Perceptual Test–3rd Edition (MVPT) was used to assess the visual perception skills of spatial relationships, visual discrimination, figure-ground, visual closure, and visual memory without requiring the use of motor skills.^9,32^ The stimulus booklet was centered in front of the participant, and instructions were read verbatim for each subsection. Responses were recorded as correct or incorrect. Raw scores were translated into standard scores based on participant age at the time of testing, where higher scores reflect better function.

The Apples Test is a cancellation task used to assess speed and accuracy of visual attention during scanning.^33^ The task has 150 apples as target items, where 100 apples have an opening on the right or left, and 50 apples are complete. Participants sat in a chair facing the table and the task sheet was placed directly at the center in front of them. Instructions were given to “cross out all the full apples and ignore the apples with a bite taken out of them” as fast as possible and to tell the examiner when they were finished. Participants were provided with a marker pen and the timer was started as soon as the first target was crossed out. The timer was stopped when the participant stated they were done, or at a maximum time limit of 300 seconds. The total number of targets identified and total time taken to complete the assessment were recorded.

Jerry John's Basic Reading Inventory (BRI) was designed to assess reading fluency and word recognition and is familiar in most educational contexts.^34^ Grade reading level was measured using Jerry John’s Basic Reading Inventory through assessed performance on a word list and a word passage compared with actual grade level and reading speed in words per minute. Participants were provided with a list of words from Jerry John’s Basic Reading Inventory corresponding to the child's actual grade level and were instructed to read at their own pace. Participants who read 19-20 words correctly were considered “independent” at that grade level and were advanced a grade to attempt a more challenging word list. Those who correctly read 18 or fewer words were provided a list from the lower grade level. This process continued until the participant reached their “independent” reading level, to a maximum of 2 grades above or below actual grade level. The same procedure was applied to the reading passage task where miscues were defined as word omissions, substitutions, insertions, repeated mispronunciations, or skipping an entire line. A score of 0-3 miscues indicated “independent” reading level at the tested grade. Reading passages were timed, and words per minute were calculated.

### Statistical Analysis

Statistical analyses were completed in Jamovi (version 2.3).^35^ Age and sex distributions between groups were assessed using *t* test and a χ^2^ test of independence. To explore differences between perinatal arterial ischemic stroke and typically developing controls in perceptual performance, data normality was first assessed using the Shapiro-Wilk test, and Levene′s test was used to identify homogeneity of variances. Student *t* tests were used in the case of normally distributed data with equal variance. One-way analysis of variance was used to identify Motor-Free Visual Perceptual Test performance differences in children with right and left hemisphere strokes compared to controls, with Fisher test used in cases of unequal variances and small sample sizes. The Mann-Whitney *U* test was applied where data were not normally distributed (*P* < .05), and the Welch *t* test was used in cases of normal distribution but unequal variance between groups (*P* < .05). Pearson correlation was used to identify associations between white matter microstructure variables and functional test performance in the case of normally distributed data. When data were not normally distributed, a Spearman rank correlation was used. Correlations were partially adjusted for age to account for white matter microstructural changes during development.^36^ The Benjamini-Hotchberg method was used to control for multiple comparisons with a false discovery rate of 0.05.^37^

## Results

### Demographics

Fifty-three participants were recruited (Table 1). Twenty-seven typically developing controls and 26 participants with perinatal arterial ischemic stroke completed perceptual testing; 1 child with perinatal arterial ischemic stroke and 3 typically developing children declined to complete the reading task. Twenty-three participants from each group successfully completed the MRI. Age (*t*_51_ = −0.315, *P* = .754) and sex (χ^2^_1(N_ _=_ _53)_ *=* 0.925, *P* = .336) were comparable between groups. Of 24 perinatal arterial ischemic stroke participants who had completed visual field testing, 2 had been diagnosed with visual field deficits. Five children with perinatal arterial ischemic stroke and 3 typically developing controls had diagnoses of ADHD that were managed with medication.

**Table 1.** Participant Demographic and Anatomical Characteristics.

Characteristics by participant group	PAIS (n = 26)	TDC (n = 27)
Age, y, mean ± SD (min-max)	11.9 ± 3.0 (7.0-18.0)	11.6 ± 3.3 (7.0-17.0)
Sex, n (%)		
Male	12 (46.2)	17 (63.0)
Female	14 (53.8)	10 (37.0)
Stroke hemisphere, n (%)		
Left	18 (69.2)	**–**
Right	8 (30.8)	**–**
Lesion location, n (%)		
Proximal M1	6 (23.1)	**–**
Distal M1	7 (26.9)	**–**
Posterior trunk	6 (23.1)	**–**
Anterior trunk	2 (7.7)	**–**
Lenticulostriate	4 (15.4)	**–**
Other	1 (3.8)	**–**
Sensorimotor impairment, n (%)		
Present	14 (53.8)	**–**
Absent	12 (46.2)	**–**

Abbreviations: PAIS, perinatal arterial ischemic stroke; SD, standard deviation; TDC, typically developing controls.

### Group Comparisons of Visuoperceptual Task Performance

#### Motor-Free Visual Perceptual Test

**Figure 2.**
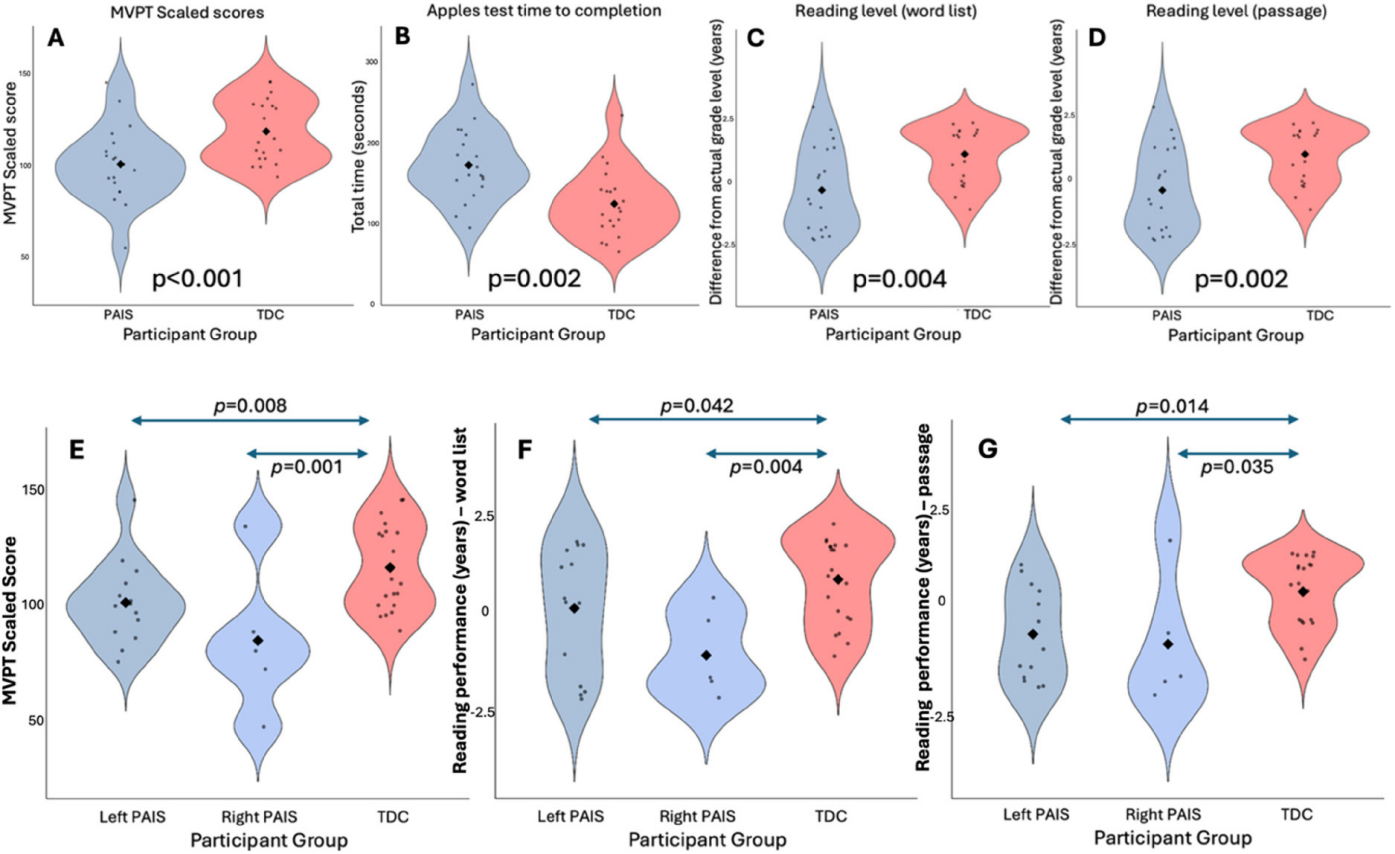
Top: Violin plots illustrating differences in task performance between stroke (blue) and control (red) participants between scores on the Motor-Free Visual Perceptual Test–3rd Edition (MVPT) (A), time taken to complete the Apples Test (B), and reading performance for a word list (C) or passage (D) compared to actual grade level on the Jerry John's Basic Reading Inventory. Bottom: Violin plots illustrating differences in task performance between left (dark blue) and right hemisphere stroke (light blue), and control (red) participants illustrating differences between scores on the Motor-Free Visual Perceptual Test–3rd Edition (MVPT) (E) and reading performance for a word list (F) or passage (G) compared to actual grade level on the Jerry John's Basic Reading Inventory. PAIS, perinatal arterial ischemic stroke; TDC, typically developing child(ren).

Children with perinatal arterial ischemic stroke had poorer performance on the Motor-Free Visual Perceptual Test (*t*_52_ = 4.169, *P* < .001, *d* = 1.135) compared with typically developing controls (Figure 2). Children with right hemisphere lesions performed worse than children with left hemisphere lesions when compared with typically developing controls (*F*_(2, 17.4)_ = 7.890, *P* = .004) (Figure 2). There were no significant sex differences in task performance. There were no differences in performance in perinatal arterial ischemic stroke participants with versus without sensorimotor impairments.

#### The Apples Test

For the Apples Test, there was no difference in the number of targets identified between groups; however, children with perinatal arterial ischemic stroke required more time to complete the scanning task; *U*(N_AIS_ = 27, N_TDC_ = 26) = 174.0, *P* = .002, mean difference of 41 seconds (Figure 2). Children with right hemisphere lesions had greater variance than typically developing controls and perinatal arterial ischemic stroke participants with left hemisphere lesions (*F*_(2, 50)_ = 4.240, *P* = .020). There was no significant difference in scanning time for perinatal arterial ischemic stroke participants based on lesion side. There were no sex differences for either total target identification or scanning speed. No differences in performance were present in perinatal arterial ischemic stroke participants with versus without sensorimotor impairments.

#### Jerry John’s Basic Reading Inventory

For assessed reading levels, compared with typically developing controls, children with perinatal arterial ischemic stroke had lower reading level for word lists, *U*(N_AIS_ = 26, N_TDC_ = 24) = 165.00 (*P* = .004), and passages of text, *U*(N_AIS_ = 26, N_TDC_ = 24) = 157.50 (*P* = .002) (Figure 2). There was no difference in reading speed between groups. Males performed better than females when reading a list of words, *U*(N_Male_ = 28, N_Female_ = 22) = 210.50 (*P* = .052). Perinatal arterial ischemic stroke participants with right hemisphere lesions had greater difficulty with reading word lists (*F*_(2, 18.6)_ = 8.161, *P* = .003) and passages (*F*_(2, 17.1)_ = 5.610, *P* = .013) compared with controls than participants with left hemisphere lesions (Figure 2). There were no significant differences in reading speed between participants with perinatal arterial ischemic stroke with right and left hemisphere lesions. There were no sex differences in reading speed or when reading a passage. There were no differences in reading performance in participants with perinatal arterial ischemic stroke with versus without sensorimotor impairments.

### Correlations Between Performance on Different Perceptual Tasks

#### Arterial ischemic stroke

Motor-Free Visual Perceptual Test scores were positively correlated with the number of targets identified in the Apples Test (*r*_s(25)_ = 0.604, *P* = .001) and reading speed (*r*_21_ = 0.593, *P* = .001). Reading performance on a word list was positively correlated with performance reading a passage (*r*_s(24)_ = 0.879, *P* = .001) (Table 2).

**Table 2.** Correlation Matrix Showing Correlations Between Functional Task Performance in Children With PAIS and TDC.^a^

PAIS					
	Apples total	Apples time (seconds)	BRI word list	BRI passage	BRI speed (WPM)
MVPT	*r*_s(25)_ = 0.604 *P* = .001**	*r*_25_ = 0.319	*r*_s(24)_ = 0.440	*r*_s(24)_ = 0.423	*r*_21_ = 0.593 *P* = .001**
Apples total		*r*_s(25)_ = 0.437	*r*_s(24)_ = 0.447	*r*_s(24)_ = 0.484	*r*_s(21)_ = 0.502
Apples time (seconds)			*r*_s(24)_ = 0.375	*r*_s(24)_ = 0.393	*r*_21_ = 0.060
BRI word list				*r*_s(24)_ = 0.879 *P* = .001**	*r*_s(21)_ = 0.403
BRI passage					*r*_(21)_ = 0.395

TDC					
	Apples total	Apples time (seconds)	BRI word list	BRI passage	BRI speed (WPM)
MVPT	*r*_s(25)_ = 0.360	*r*_s(25)_ = −0.188	*r*_s(23)_ = 0.290	*r*_s(23)_ = 0.231	*r*_23_ = −0.087
Apples total		*r*_s(25)_ = 0.046	*r*_s(23)_ = 0.002	*r*_s(23)_ = −0.154	*r*_s(22)_ = −0.078
Apples time (seconds)			*r*_s(21)_ = 0.331	*r*_s(21)_ = 0.304	*r*_22_ = −0.223
BRI word list				*r*_s(22)_ = 0.739 *P* = .001**	*r*_s(22)_ = 0.018
BRI passage					*r*_(22)_ = −0.061

Abbreviations: BRI, Jerry John's Basic Reading Inventory; MVPT , Motor-Free Visual Perceptual Test–3rd edition; PAIS, perinatal arterial ischemic stroke; TDC, typically developing child(ren); WPM, words per minute.

^a^ Correlation matrix showing correlations between functional task performance in PAIS (top) and TDC (bottom).

#### Typically developing controls

Performance on reading a word list was significantly correlated with performance reading a passage (*r*_s(22)_ = 0.739, *P* = .001). There were no other significant correlations between performance on visuospatial and reading assessments (Table 2).

### Perceptual Task Performance and Associations With White Matter Microstructure

#### Motor-Free Visual Perceptual Test

In children with perinatal arterial ischemic stroke, scaled scores on the Motor-Free Visual Perceptual Test were not correlated with white matter microstructure of the lesioned hemisphere. In the nonlesioned hemisphere, Motor-Free Visual Perceptual Test scaled scores were negatively correlated with MD (*r*_21_ = −0.508, *P* = .014) (Table 3). In the typically developing control participant group, there were no correlations between white matter metrics and scaled scores on the Motor-Free Visual Perceptual Test in either the dominant or nondominant hemispheres.

**Table 3.** Partial Correlations, Adjusted for Age, Comparing Extracted White Matter Metrics and Task Performance in PAIS.

	Lesioned Hemisphere				Nonlesioned hemisphere			
	Fractional anisotropy	Mean diffusivity	Axial diffusivity	Radial diffusivity	Fractional anisotropy	Mean diffusivity	Axial diffusivity	Radial diffusivity
MVPT	*r*_21_ = 0.287	*r*_s(21)_ = −0.199	*r*_s(21)_ = −0.184	*r*_s(21)_ = −0.290	*r*_21_ = 0.102	*r*_21_ = −0.508	*r*_21_ = −0.391	*r*_21_ = −0.400
Apples total	*r*_s(21)_ = 0.181	*r*_s(21)_ = 0.248	*r*_s(21)_ = 0.154	*r*_s(21)_ = 0.194	*r*_s(21)_ = −0.290	*r*_s(21)_ = 0.009	*r*_s(21)_ = −0.013	*r*_s(21)_ = 0.091
Apples time	*r*_21_ = −0.173	*r*_s(21)_ = 0.148	*r*_s(21)_ = 0.044	*r*_s(21)_ = 0.279	*r*_s(21)_ = −0.292	*r*_s(21)_ = 0.028	*r*_s(21)_ = −0.231	*r*_s(21)_ = 0.077
BRI word list	*r*_s(20)_ = 0.475	*r*_s(20)_ = −0.078	*r*_s(20)_ = 0.010	*r*_s(20)_ = −0.248	*r*_s(20)_ = 0.117	*r*_s(21)_ = −0.310	*r*_s(20)_ = −0.092	*r*_s(20)_ = −0.307
BRI passage	*r*_s(20)_ = 0.366	*r*_s(20)_ = −0.090	*r*_s(20)_ = 0.009	*r*_s(20)_ = −0.184	*r*_s(20)_ = 0.057	*r*_s(21)_ = −0.267	*r*_s(20)_ = −0.159	*r*_s(20)_ = −0.288
BRI speed (WPM)	*r*_18_ = 0.379	*r*_s(18)_ = −0.373	*r*_s(18)_ = −0.361	*r*_s(18)_ = −0.388	*r*_18_ = 0.326	*r*_18_ = −0.628	*r*_18_ = −0.382	*r*_18_ = −0.595

Abbreviations: BRI, Jerry John's Basic Reading Inventory; MVPT, Motor-Free Visual Perceptual Test–3rd edition; WPM, words per minute.

#### The Apples Test

For perinatal arterial ischemic stroke participants, white matter microstructure of the lesioned or nonlesioned hemisphere was not correlated with the total number of apples correctly identified or time taken to complete the task (Table 3). There were no significant correlations between scores on the Apples Test and white matter metrics in typically developing controls in either hemisphere.

#### Jerry John’s Basic Reading Inventory

**Figure 3.**
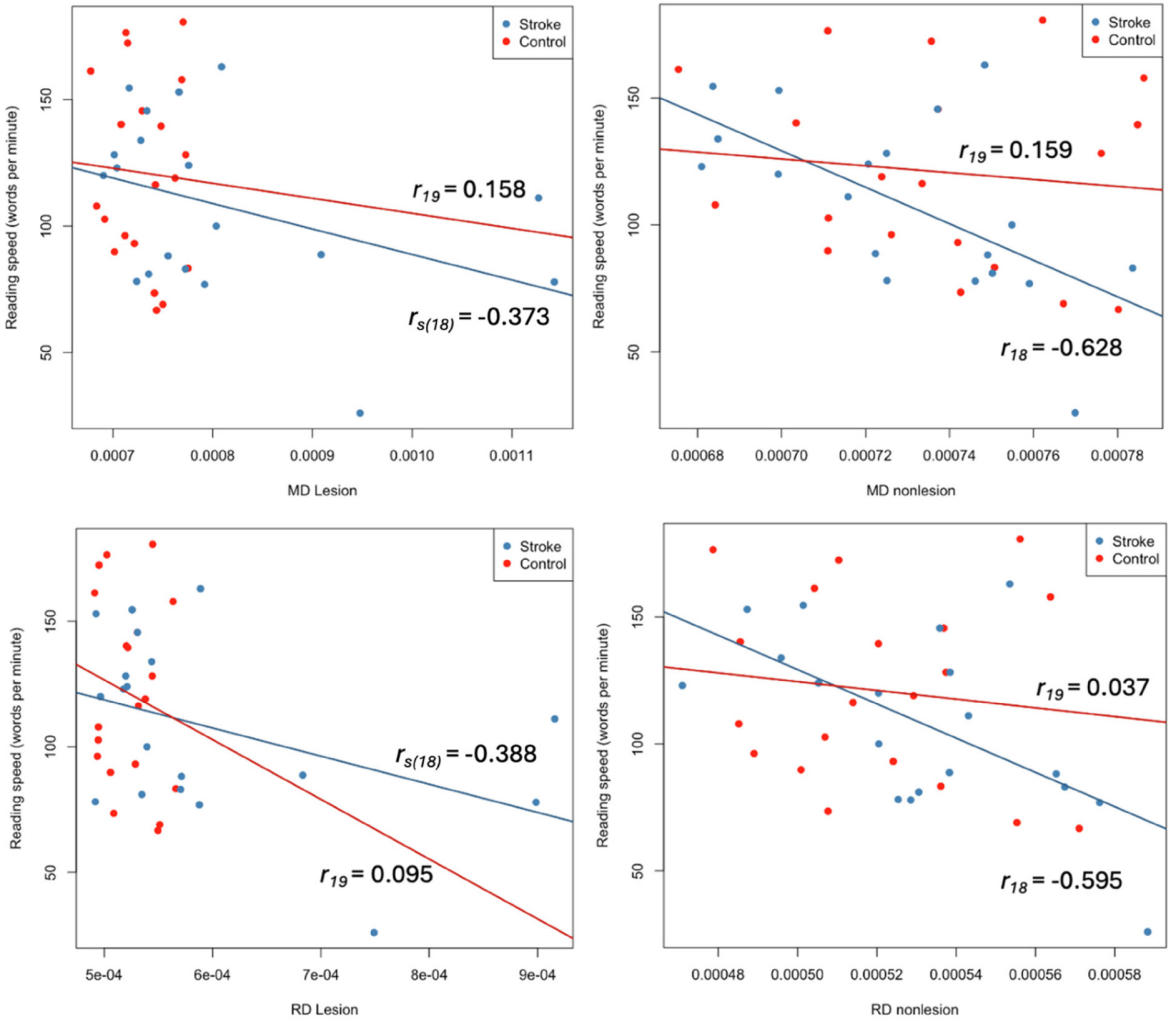
Scatter plots of stroke (blue) and control (red) participants illustrating correlations between reading speed (words per minute) and white matter metrics of MD (Mean diffusivity), RD (radial diffusivity – bottom) in the lesioned/non-dominant (left) and nonlesioned/dominant (right) hemispheres. Lines of best fit are presented for stroke (blue) and control (red) groups. Pearson or Spearman correlations and significant *P* values are listed.

In the nonlesioned hemisphere of perinatal arterial ischemic stroke participants, reading speed was negatively correlated with MD (*r*_18_ = −0.628, *P* = .005) and RD (*r*_18_ = −0.595, *P* = .009); however, these associations did not reach significance after correcting for multiple comparisons (Figure 3). White matter microstructure was not significantly correlated with reading ability or speed in the typically developing control group.

## Discussion

We used visuoperceptual testing and white matter tractography to assess visual function in a group of children with perinatal arterial ischemic stroke compared with typically developing controls as well as explore structure-function associations. Results of this study identified poorer visuoperceptual task performance in children with perinatal arterial ischemic stroke, with and without the presence of motor impairment, compared with their age- and sex-matched peers. In perinatal arterial ischemic stroke participants, performance on one visuoperceptual task was more likely to be correlated with performance on other visuoperceptual tasks, which was not observed in our typically developing control group. Extracted microstructure metrics from white matter reconstructions of the optic tracts and optic radiations did not meet statistical significance when correlated with visuoperceptual task performance. The pattern of associations between white matter microstructure and task performance suggested group-specific trends that may benefit from future investigation with a larger sample size. Specifically, perinatal arterial ischemic stroke microstructure associated with performance on the Motor-Free Visual Perceptual Test and reading speed, and typically developing control microstructure was more closely linked with the ability to read a list of words.

### Task Performance Between Groups

Clinically, it is important to note that children with perinatal arterial ischemic stroke performed worse on standardized tasks of visuoperceptual skills and reading ability and were significantly slower on scanning during the Apples Test. Compared with age- and sex-matched peers, more children with perinatal arterial ischemic stroke missed a significant number of targets, which is in line with previous assessment of scanning abilities in children with cerebral palsy.^17,38^ As the current literature has focused on visuoperceptual performance in children with cerebral palsy, the finding that participants with perinatal arterial ischemic stroke without residual motor impairment are also trailing behind their typically developing peers is clinically relevant. Results suggest that health care providers should screen for visuoperceptual impairments in all children with perinatal arterial ischemic stroke, ideally before they reach school age, to enable early intervention.

Although limited by a small sample size, children with right hemisphere lesions had the lowest scores on the Motor-Free Visual Perceptual Test, reading performance, and the Apples Test. In studies of cerebral palsy and perinatal stroke, children with right hemisphere lesions present with worse attention to the contralateral environment^38–41^ and lower Motor-Free Visual Perceptual Test scores.^10^ Despite our understanding of this lateralization of visuospatial attention toward the right hemisphere, lesion side is not always considered in studies of visuoperceptual skills such as object recognition, figure-ground, or visual closure in children with cerebral palsy^42^ and suggests an area needing additional study. Furthermore, lateralization of function is altered in both hemispheres following early unilateral brain injury,^13^ limiting the anticipated effects of traditional hemisphere-dependent effects such as handedness.

Another interesting finding in the perinatal arterial ischemic stroke group was that performance levels across perceptual, visuospatial, and reading tasks were positively correlated, where poorer performance on one task was likely to be linked to poorer performance on the other tasks, and vice versa. In the typically developing control group, other than a positive correlation between ability to read lists and passages, visuoperceptual skills were not linked between the various tasks, suggesting that poor performance on one element of visuoperceptual function is not necessarily linked to poor performance in other areas of visuoperceptual function. This suggests that visuoperceptual function or dysfunction may affect children with perinatal arterial ischemic stroke more globally than their peers. Clinically, the interconnectedness of visuoperceptual abilities in participants with perinatal arterial ischemic stroke suggests that screening using one tool, such as the Motor-Free Visual Perceptual Test, may provide information as to whether additional screening should be conducted. Additionally, global visuoperceptual dysfunction could contribute to challenges across numerous daily activities such as navigating busy environments, reading and academic performance, or difficulty recognizing faces that could lead to decreased social participation.

### Task Performance and Correlations With White Matter Microstructure

Visuoperceptual function and white matter microstructure of the afferent visual pathways were not correlated in either experimental group after correcting for multiple comparisons. High variability in our perinatal arterial ischemic stroke sample may have contributed to this lack of clear findings. Recruitment of motor-intact participants in this study compared to a previous study^20^ may have contributed to this variability. The patterns that emerged in the perinatal arterial ischemic stroke group, with stronger associations in the nonlesioned hemisphere, are suggestive of bihemispheric developmental alterations in children with perinatal stroke that has been previously documented in other brain areas.^20,43–46^ Larger sample sizes in future studies may be able to clarify whether statistically significant correlations are present between the assessed factors.

### Limitations

Results of this study were limited by the sample size, calling for fully powered studies to confirm our findings. Future research should consider limitations we observed in the clinical assessments. A ceiling effect was seen on the Apples Test, with all or most of the 50 targets identified in the allotted 300 seconds. The Jerry John’s Basic Reading Inventory protocol required that the assessor stop after the participant achieved independence in reading at 2 above or 2 below their actual grade level, whereas some participants may have scored higher or lower if they had continued. Additionally, Jerry John’s Basic Reading Inventory assessed reading fluency only and does not adequately capture the cognitive complexities of reading comprehension. Reading comprehension may be a useful assessment in future studies. Analysis of variance was used to identify differences between right and left hemisphere lesions compared with typically developing controls. Only 8 participants had right hemisphere lesions, which decreased the power of this analysis. Two participants had known visual field deficits, which was not a sufficient sample size to compare with other participants. There were also limitations in the diffusion image analyses. Manual tractography was used for this analysis, as the variations in brain structure in the stroke participants prevented automation of this task. Manual tractography is user dependent and inherently has some user bias.^45^ Diffusion imaging and tractography are used regularly in imaging to re-create representations of white matter tracts of significance. The extracted metrics of fractional anisotropy, mean diffusivity, axial diffusivity, and radial diffusivity are all subject to the issue of crossing-fibers.^47,48^ Sampling of additional, higher-level pathways, postprimary visual cortex, could also have been informative.

## Conclusion

This study identified that children with perinatal arterial ischemic stroke—with and without motor dysfunction—are more likely to present with visuoperceptual impairment than their age- and sex-matched peers. Additional research is needed to determine clinically significant differences in participants with perinatal arterial ischemic stroke with right versus left hemisphere lesions. Exploring developmental white matter differences of additional secondary visual pathways would allow for a better understanding of the structure-function relationship between white matter and visuoperceptual skills.
